# FRAGMENTED FAMILIES OF OLDER FORCED UKRAINIAN MIGRANTS: PRELIMINARY DATA FROM A HELP CENTER IN NORTHERN ISRAEL

**DOI:** 10.1093/geroni/igac059.3104

**Published:** 2022-12-20

**Authors:** Ksenya Shulyaev, Yulia Macarenko, Tova Band-Winterstein

**Affiliations:** University of Haifa, Haifa, Hefa, Israel; Volunteer Help Center for Refugees from Ukraine, Haifa, Hefa, Israel; University of Haifa, Haifa, Hefa, Israel

## Abstract

Since the beginning of the Russia-Ukrainian war, more than 50,000 people have come to Israel from Ukraine as “repatriates” (able to acquire citizenship) or “tourists” (with a right to live in Israel during the war, without citizenship), about 30% are older adults, for whom forced migration poses serious challenges. The aim of this presentation is to describe the socio-demographic characteristics of older migrants’ fragmented families, who turn to one of the Volunteer Help Center in Northern Israel. Descriptive statistics on older adults from the database of about 900 families who received support during April-June 2022 were performed. Data revealed: 189 migrants aged 55+ (M(SD)=69.7(8.2); range 55–89); 148 (78.3%) were female; 106 (56%) had Israeli citizenship or were in the process of receiving it; 75 (40 %) were “tourists”. Among “tourists”, about half (Nf30) came to visit their adult children. “Repatriates” and “tourists” did not differ in age (F(159)=1.574, p=ns). As a rule, older adults came with family members (Nf136(72.7%)). However among “tourists” 41.9% came alone and only 17.1% among “repatriates” (X=13.374, p < 0.001). Older adults were mostly accompanied by daughters (Nf80(58.8%)), usually without husbands, in 127 fragmented families (78.2%) were children under 18. Only 15% came with a spouse (half of the men, 5.5% of women), and five came with mothers. This preliminary data draws attention to the unique family fragmented structure of older displaced people, as this should be considered when developing an assistance program.

